# Supplementary material for: Modification Strategy of D-leucine Residue Addition on a Novel Peptide from Odorrana schmackeri, with Enhanced Bioactivity and In Vivo Efficacy
Source: Toxins (Basel). 2021 Aug 31;13(9):611. doi: 10.3390/toxins13090611 (PMC8473181; doi:10.3390/toxins13090611)
Supplement: Supplementary file 1 [file toxins-13-00611-s001.zip › toxins-1299038-supplementary.pdf]

Modification Strategy of D-leucine Residue Addition on a Novel Peptide from *Odorrana schmackeri*, with Enhanced Bioactivity and In Vivo Efficacy

Supplementary Materials

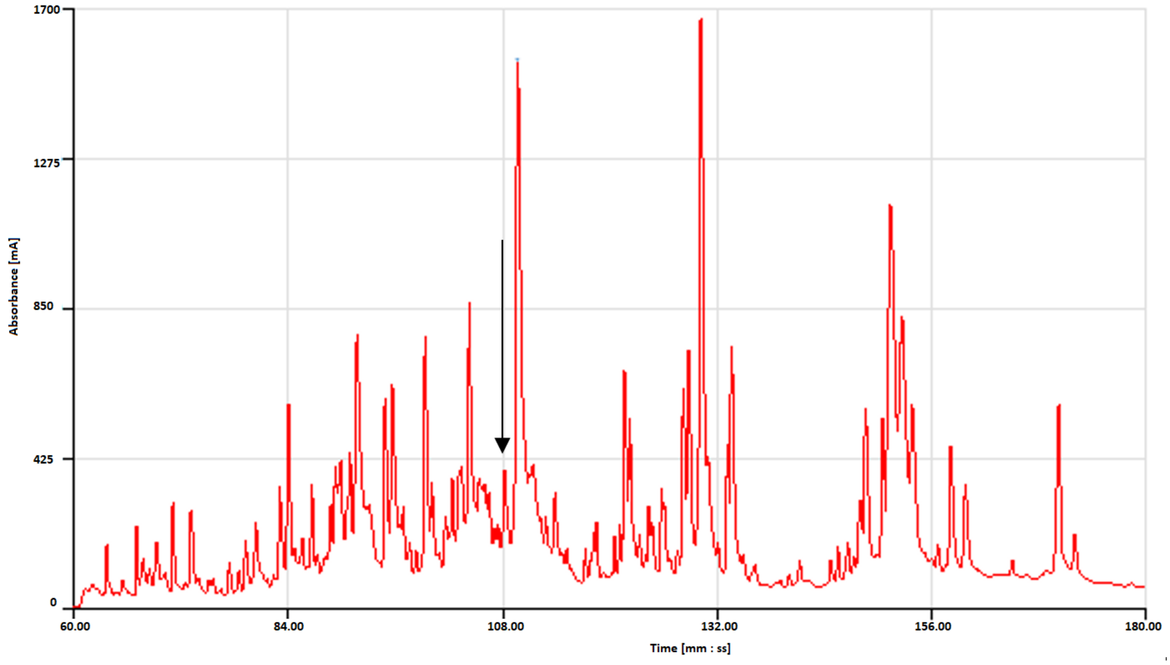

(A)

#1	b(1+)	b(2+)	Seq.	y(1+)	y(2+)	#2
1	148.07570	74.54149	F			23
2	245.12847	123.06787	P	2371.37175	1186.18951	22
3	358.21254	179.60991	L	2274.31898	1137.66313	21
4	471.29661	236.15194	I	2161.23491	1081.12109	20
5	542.33373	271.67050	A	2048.15084	1024.57906	19
6	629.36576	315.18652	S	1977.11372	989.06050	18
7	742.44983	371.72855	L	1890.08169	945.54448	17
8	813.48695	407.24711	A	1776.99762	889.00245	16
9	870.50842	435.75785	G	1705.96050	853.48389	15
10	984.55135	492.77931	N	1648.93903	824.97315	14
11	1083.61977	542.31352	V	1534.89610	767.95169	13
12	1182.68819	591.84773	V	1435.82768	718.41748	12
13	1279.74096	640.37412	P	1336.75926	668.88327	11
14	1407.83593	704.42160	K	1239.70649	620.35688	10
15	1520.92000	760.96364	I	1111.61152	556.30940	9
16	1667.98842	834.49785	F	998.52745	499.76736	8
17	1770.99761	886.00244	C	851.45903	426.23315	7
18	1899.09258	950.04993	K	748.44984	374.72856	6
19	2012.17665	1006.59196	I	620.35487	310.68107	5
20	2113.22433	1057.11580	T	507.27080	254.13904	4
21	2241.31930	1121.16329	K	406.22312	203.61520	3
22	2397.42042	1199.21385	R	278.12815	139.56771	2
23			C	122.02703	61.51715	1

(B)

Figure S1. Isolation and identification of B1OS from frog skin secretion. (**A**) RP-HPLC chromatogram of the skin secretion of *Odorrana schmackeri* detected at the wavelength of 214 nm. The elution peak of B1OS is indicated by an arrow. (**B**) Predicted b-ion and y-ion MS/MS fragment ion series (singly- and doubly-charged) of B1OS. The actual ions detected in MS/MS spectra are coloured.

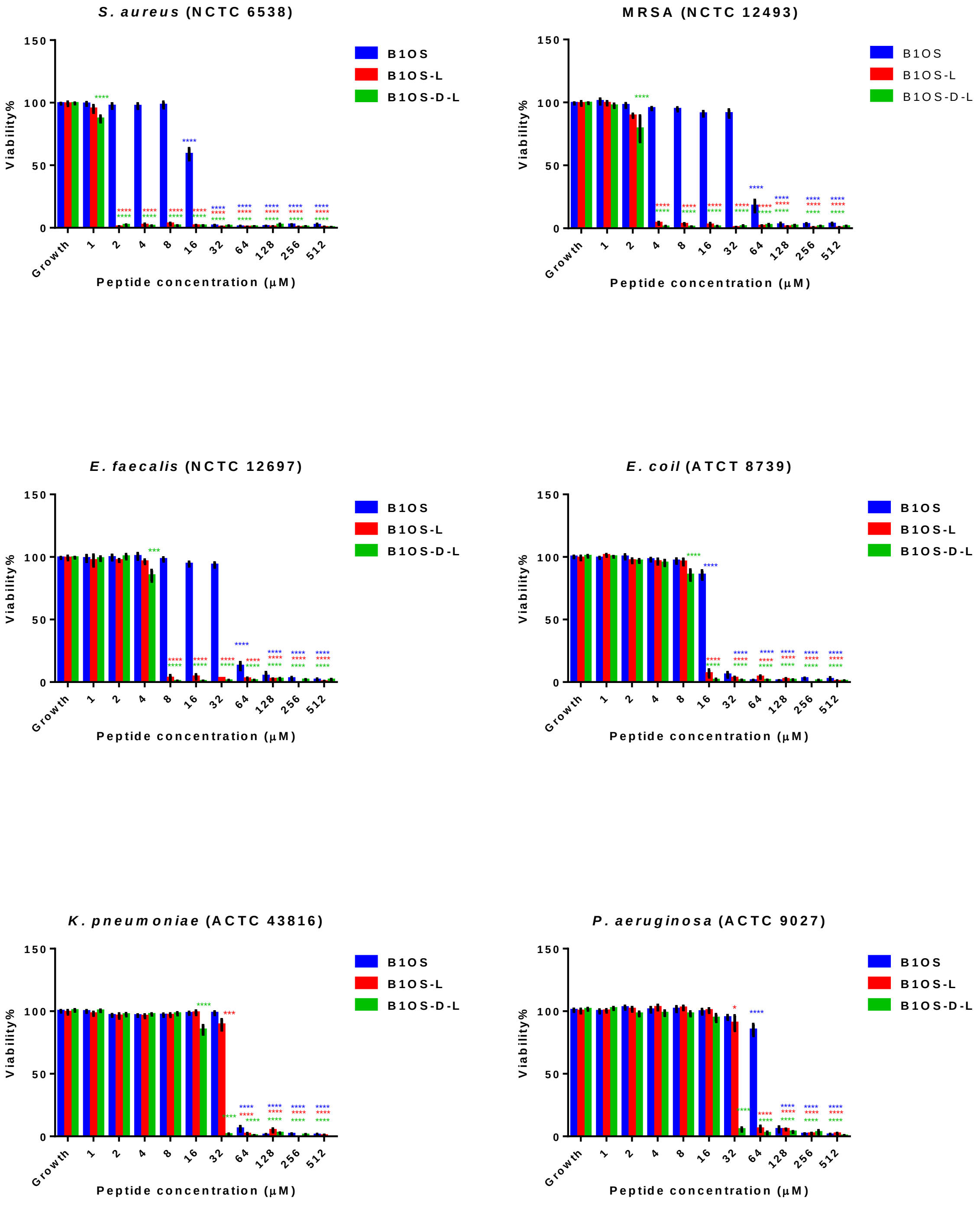

Figure S2. The viability of six tested microorganisms in the treatment of B1OS, B1OS-L and B1OS-D-L at the concentration from 1 to 512 μM. The error bar represents the means ± standard deviation of three independent experiments. The statistical analysis was calculated by ordinary two-way ANOVA, and the statistical significance is indicated as * ($p < 0.05$), ** ($p < 0.01$), *** ($p < 0.001$) and **** ($p < 0.0001$) versus growth control group (no treatment).

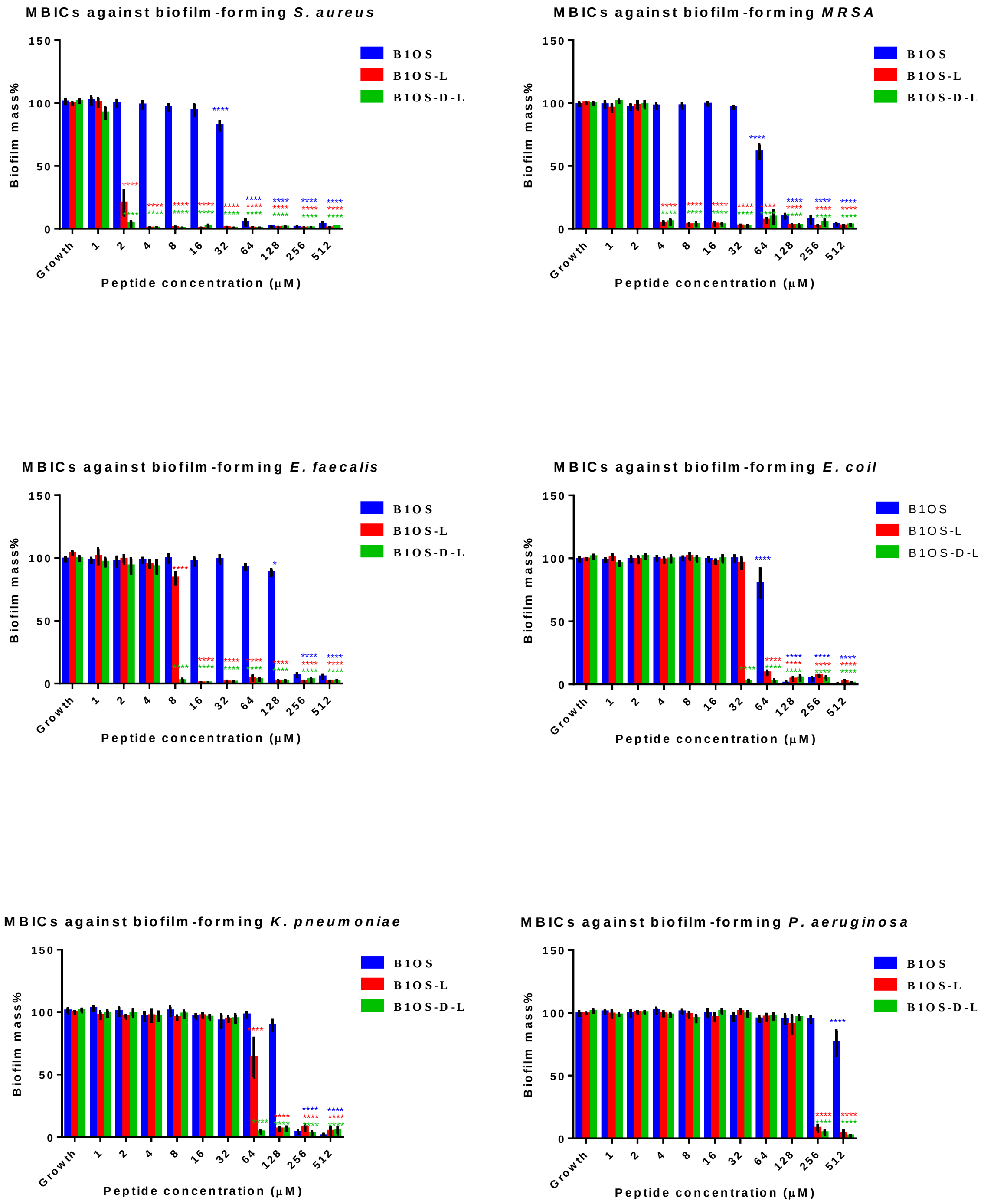

Figure S3. The percentage of biofilm mass of six microorganisms after the treatment of B1OS, B1OS-L and B1OS-D-L in the biofilm inhibition assays (MBIC). The error bar represents the means ± standard deviation of three independent experiments. The statistical analysis was calculated by ordinary two-way ANOVA, and the statistical significance is indicated as * ($p < 0.05$), ** ($p < 0.01$), *** ($p < 0.001$) and **** ($p < 0.0001$) versus growth control group (no treatment).

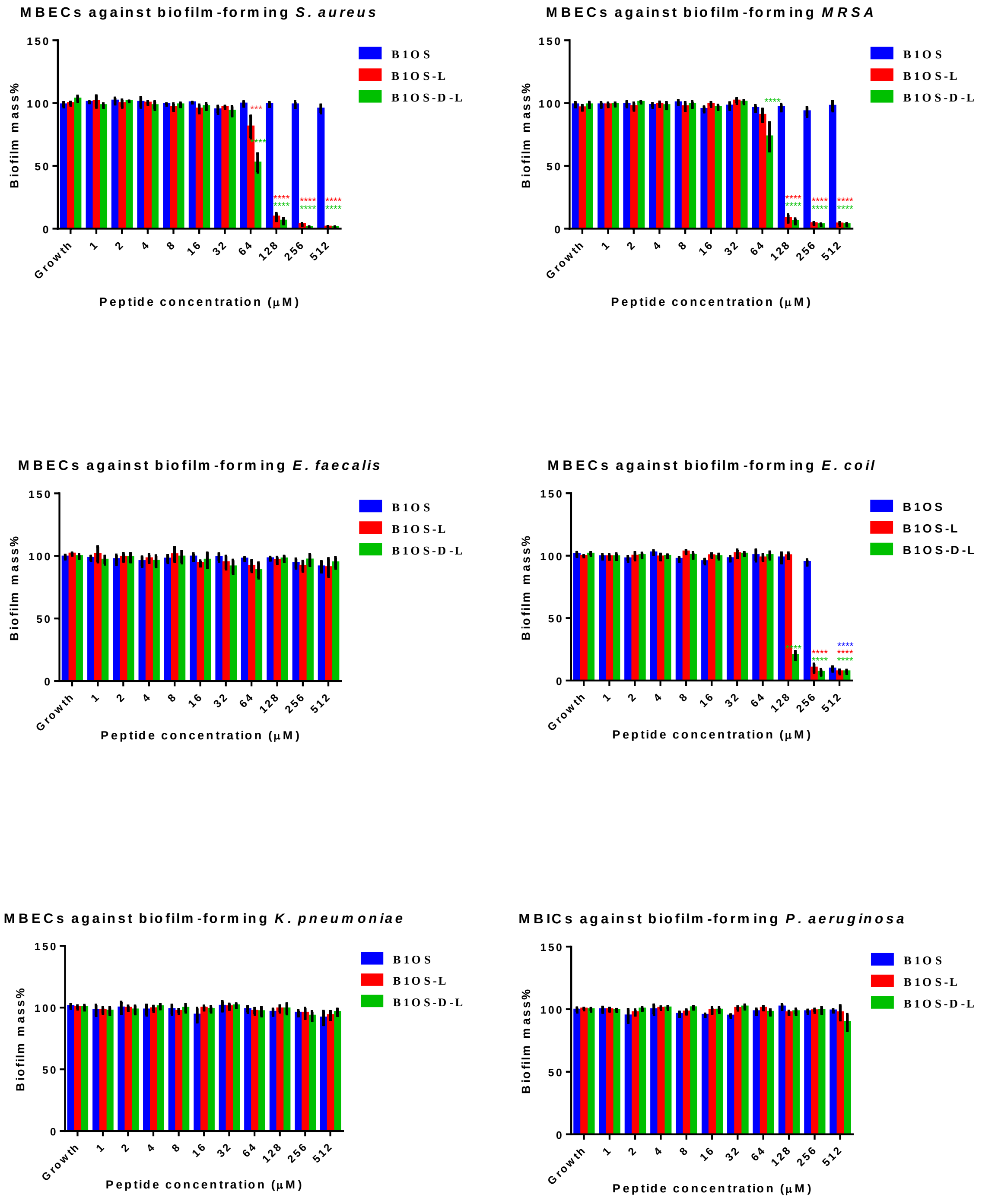

Figure S4. The percentage of biofilm mass of six microorganisms after the treatment of B1OS, B1OS-L and B1OS-D-L in the biofilm eradication assays (MBEC). The error bar represents the means ± standard deviation of three independent experiments. The statistical analysis was calculated by ordinary two-way ANOVA and the statistical significance is indicated as *** ($p < 0.001$) and **** ($p < 0.0001$) versus growth control group (no treatment).

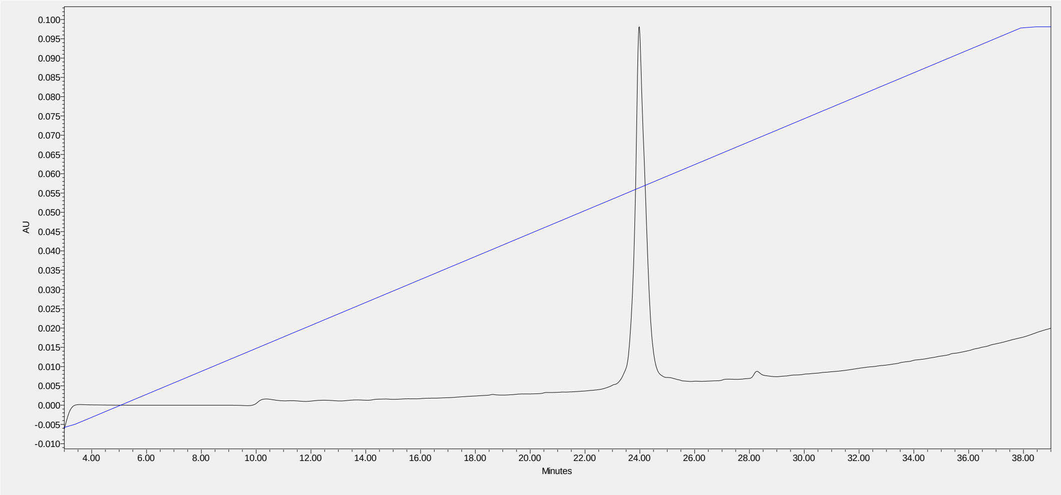

(A)

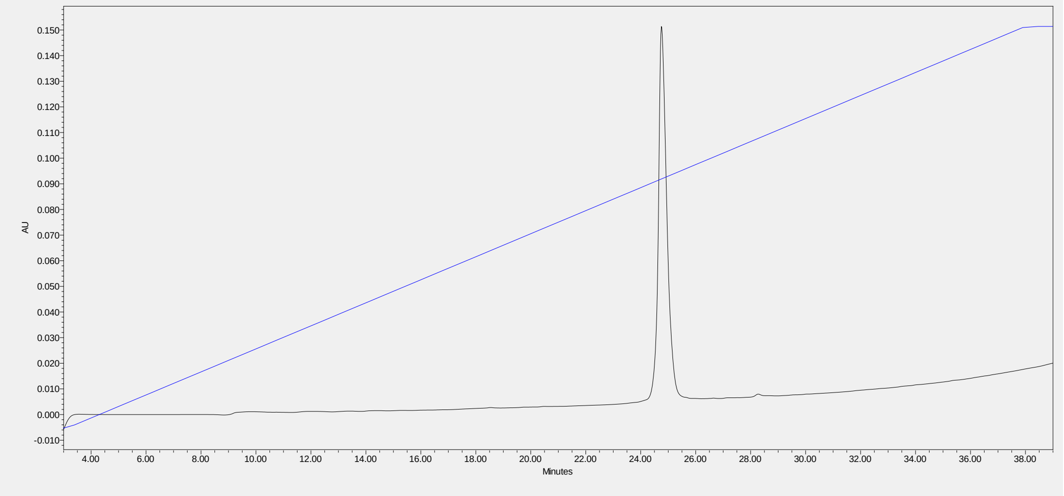

(B)

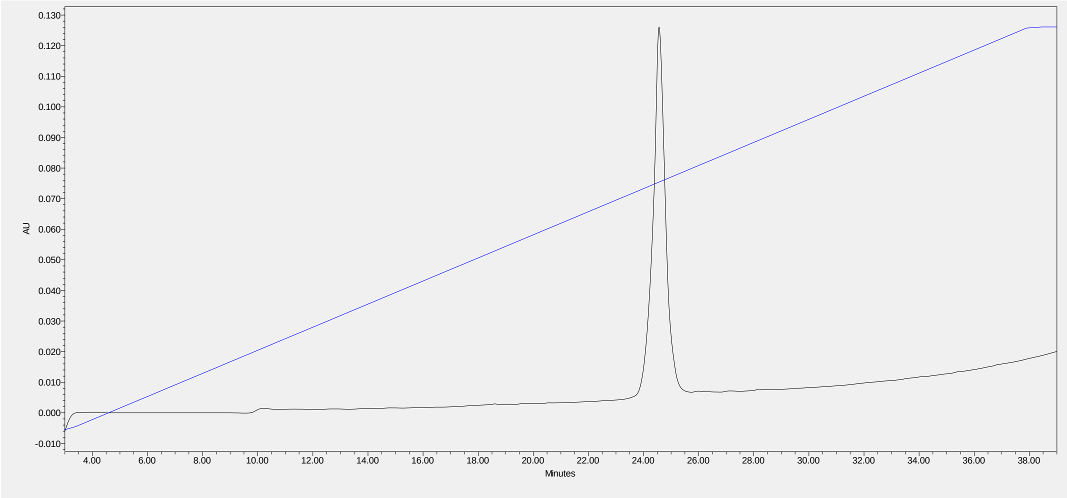

(C)

Figure S5. RP-HPLC chromatogram of purified peptides B1OS (**A**), B1OS-L (**B**) and B1OS-D-L (**C**) monitored at 214 nm. The acetonitrile gradient is indicated by solid line. The arrow indicted the retention time of the pure fragment of the peptide.

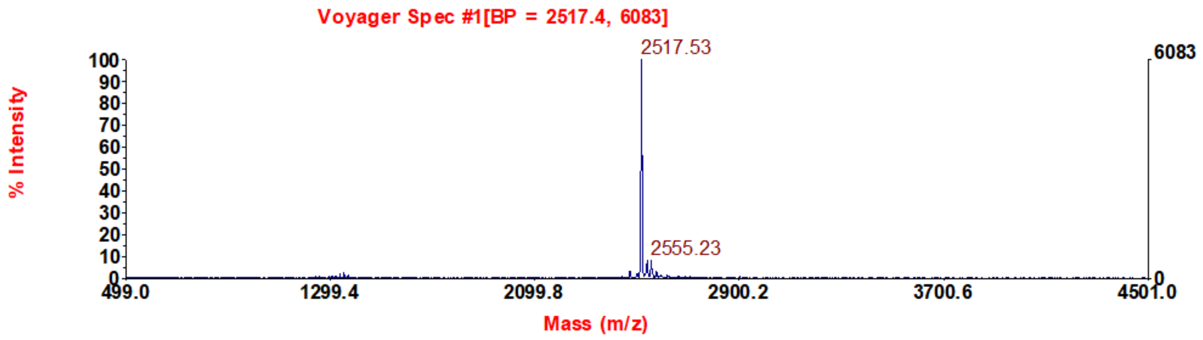

(A)

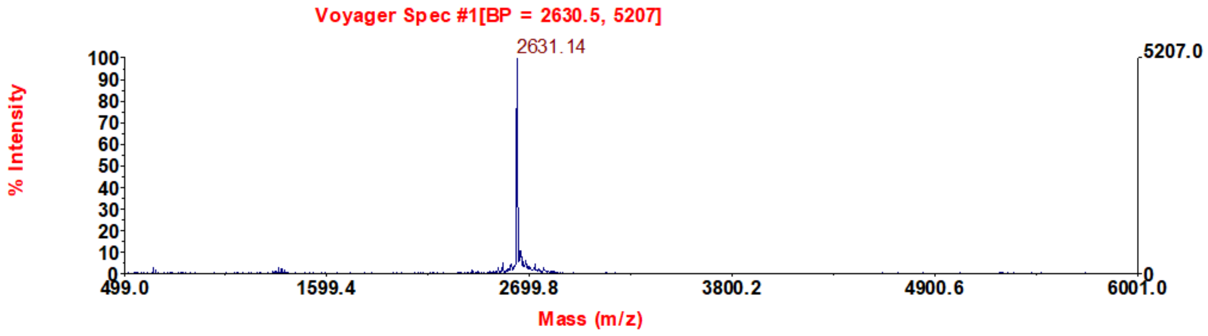

(B)

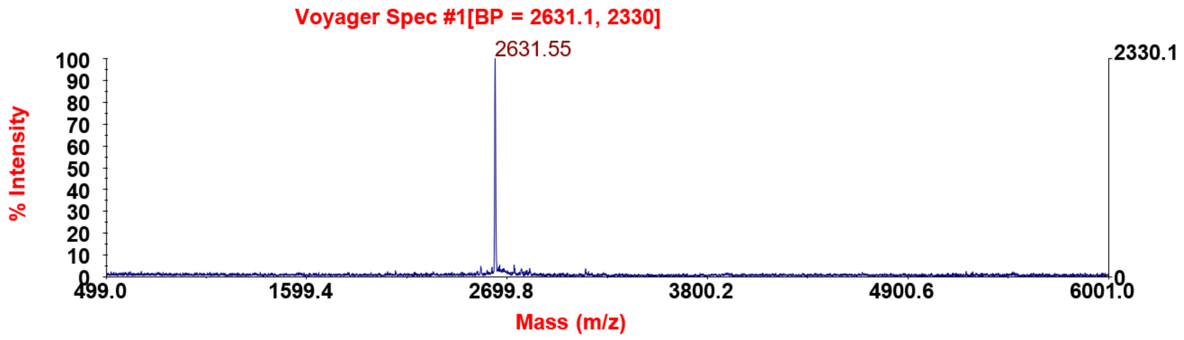

(C)

Figure S6. MALDI-TOF mass spectra of purified peptides B1OS(**A**), B1OS-L(**B**) and B1OS-D-L (**C**). The abscissa represents the region of mass-to-charge ratio (m/z). The ordinate indicates the percentage of signal intensity. BP means base peak.
